# Oral Health Knowledge, Attitudes, and Learned Clinical Skills in Pediatric Medicine Residents and Nurse Practitioner Students: A Pre-Post Design

**DOI:** 10.3390/healthcare12181807

**Published:** 2024-09-10

**Authors:** Laurie Love, Francisco Ramos-Gomez, Janni J. Kinsler, Cristina Cabrera-Mino, Cambria Garell, Nancy A. Pike

**Affiliations:** 1 School of Nursing, University of California Los Angeles, Los Angeles, CA 90095, USA; ccabreram@sonnet.ucla.edu (C.C.-M.); napike@uci.edu (N.A.P.); 2 Division of Pediatric Dentistry, School of Dentistry, University of California Los Angeles, Los Angeles, CA 90095, USA; frg@dentistry.ucla.edu (F.R.-G.); jnhaiem@dentistry.ucla.edu (J.J.K.); 3 Department of Pediatrics, UCLAHealth, Los Angeles, CA 90095, USA; cgarell@mednet.ucla.edu; 4 Sue & Bill Gross School of Nursing, University of California Irvine, Irvine, CA 92697, USA

**Keywords:** dental caries, oral health, interdisciplinary, nurse practitioner, physicians

## Abstract

(1) Background/Objective: California has one of the highest rates of pediatric dental caries in the nation. One way to combat this problem is through non-dental provider training programs that focus on prevention. However, there are limited data on healthcare provider training program integration and evaluation of oral health curricula focused on prevention of early childhood caries. This study will assess the change in healthcare providers’ attitudes, knowledge, and skills by implementing an interprofessional educational (IPE) oral health curriculum in medicine and nurse practitioner programs at one university in Southern California. (2) Methods: A mixed method design was employed using a pre- and post-educational survey, and end-of-program focus group interviews. Descriptive statistics and paired *t*-tests were used to assess group differences and thematic analyses for the focus groups. (3) Results: A total of 81 students (14 pediatric medicine residents, 18 pediatric, and 49 family nurse practitioners) completed the curriculum and surveys. Attitudes related to oral hygiene remained unchanged, with the nurse practitioner group showing improved clinical skills (all questions; *p* < 0.021). Knowledge scores significantly improved across all groups (paired *t*-test; *p* < 0.001). All focus groups expressed the helpfulness of the educational modules, the usefulness of the skills learned, and the benefits of IPE activities. (4) Conclusion: Healthcare providers showed improved oral health knowledge and clinical skills acquired through the oral health program and can serve as a model to educate across disciplines on the prevention of early childhood caries.

## 1. Introduction

It has been over two decades since the U.S. Surgeon General released the landmark report, *Oral Health in America*, declaring early childhood caries (ECC) as a silent epidemic, the most common preventable chronic disease in children worldwide, and mainly affecting socially disadvantaged populations [1]. In the National Survey of Children’s Health 2020–2021, California was rated as one of the worst states in the nation for pediatric dental disease [2,3]. Many children from low socioeconomic status homes have limited access to routine dental care, fresh fruits and vegetables, and fluoridated water. As oral health disparities persist in the United States, academic dentistry has joined forces with healthcare providers to tackle this public health concern [4]. This has led to national organizations (e.g., the American Association of Medical Colleges, World Health Organization, and Interprofessional Education Collaborative) requiring curriculum standards for interprofessional education (IPE) for non-dental healthcare providers to deliver oral health screening and preventative treatment in the medical environment.

Multiple oral health curricular resources have been developed for use in primary care training programs [5]. *Smiles for Life* is the most comprehensive and widely used curriculum for primary care clinicians and has been endorsed by over 20 national and international organizations [6]. The Health Resources and Services Administration (HRSA) developed five key curricular content domains, which include (1) risk assessment, (2) oral health evaluation, (3) preventative interventions, (4) communication and education, and (5) interprofessional collaborative practice for oral health educational programs [7]. The curriculum should also align with the four key areas highlighted in the International Association of Pediatric Dentistry (IAPD) Bangkok Declaration [8], which include (1) raising awareness of ECC in partnership with stakeholders, including dentists, physicians, nurses, and other health professionals, (2) limiting sugar intake for children younger than two years of age, (3) brushing twice a day with an age-appropriate amount of fluoridated toothpaste, and (4) providing preventative oral health care guidance before age one through a health professional or community health worker with referrals to dental for continued care. These domains with their core competencies and key focus areas should be integrated into oral health program curricula.

Despite these national standards, healthcare provider training programs lack oral health content [9]. A survey of nurse practitioner programs identified that, though students were receiving oral health education content, faculty felt their graduates were still not adequately prepared [10]. Similar findings were found in an international study among health educators across medicine, nursing, and pharmacy students, indicating that oral health content was lacking in the curriculum [9]. There are limited data that evaluate the attitudes, knowledge, and skills of pediatric medicine residents and nurse practitioner students [11]. Therefore, this study aims to evaluate attitudes, skills, and knowledge learned by pediatric medicine residents and both pediatric and family nurse practitioner students at the University of California Los Angeles (UCLA) using the developed Care, Access, Reach, and Education in Pediatric Dentistry (CARE-PD) curriculum related to pediatric oral health prevention. We hope to provide a replicable and sustainable IPE model for use across other University of California campuses in their dentistry, medicine, pharmacy, public health, and nurse practitioner programs.

## 2. Materials and Methods

### 2.1. Design and Setting

This study used a mixed-method design, which included a pre- and post-CARE-PD curriculum survey and a focus group to evaluate in-depth knowledge, attitudes, skills, and satisfaction with the IPE program. The study was performed at the UCLA School of Dentistry and included both the schools of medicine and nursing. The UCLA institutional review board approved the study as exempt, and no consent was required from the participants.

### 2.2. Sample Selection

The sample included 2nd-year pediatric medicine residents and 1st-year pediatric nurse practitioner (PNP) and family nurse practitioner (FNP) students enrolled in courses at UCLA that contained the CARE-PD curriculum from 2021 through 2023. Receiving the CARE-PD curriculum was part of students’ coursework but the pre- and post-survey and focus group evaluation were voluntary. A total of 101 students started the study with a final sample of 81 that completed all CARE-PD curriculum and evaluation time points yielding a response rate of 80%.

### 2.3. CARE-PD Program/Oral Health Curriculum

The CARE-PD oral health education program is a continuation of the Strategic Partnership for Interprofessional Education in Pediatric Dentistry (SPICE-PD; 2015–2020) program developed to support IPE and encourage collaborative practice among dentistry, medicine, and nursing [7]. The goal was to increase access to and utilization of oral health services for underserved populations and to enhance health professionals’ ability to target children likely to be at risk for dental caries [8,12]. The CARE-PD program (2020–2025) was developed to expand the number of potential pediatric providers to include family practice. The program aims to describe and apply basic oral health strategies, risk-based diagnosis, and treatment to prevent oral disease. The same educational curriculum was used in the SPICE-PD and CARE-PD programs with the addition of a fluoride varnish application skills lab and the option of a half-day dental clinic rotation [13].

The UCLA CARE-PD curriculum consisted of the online resource *Smiles for Life*, a National Oral Health Curriculum [6] with experiential learning modules designed for undergraduate and graduate students, two and a half hours of lectures given by a pediatric dentist and an expert registered nurse in oral health care, a skills lab practicum led by a pediatric dental resident on varnish application, and a caries risk assessment assignment on a child six years of age or less [13]. Caries Management by Risk Assessment (CAMBRA) is a preventive form that can be used by health care providers in which patients are categorized by their relative risk for developing dental caries based on risk factors, including diet, oral hygiene, fluoride regimen, and past oral health history. The CAMBRA system was developed as an evidence-based approach to preventing, reversing, and treating patients with dental caries [14].

The learning objectives/expected outcomes for the CARE-PD IPE component were to (1) increase knowledge/skills/perceptions regarding IPE and practice, (2) increase knowledge/skills regarding oral health core competencies by providing anticipatory guidance to preventing dental caries, performing an oral health risk assessment, and applying fluoride varnish, and (3) increase intention to provide care to underserved and special needs populations.

The oral health curriculum was threaded through the first-year pediatric and family nurse practitioner programs, where linking oral health to existing course content was deemed appropriate. The pediatric medical residents were given the same oral health and caries risk assessment lecture content, completed the five *Smiles for Life* modules, and performed caries risk assessments on children seen during clinical rotation over eight weeks. All participants completed a fluoride varnish lab guided by a dental resident. The pre-knowledge survey was given prior to the start of the curriculum to assess baseline oral health attitudes, knowledge, and skills. The same survey was re-administered for the pediatric medicine resident group after 8 weeks and for the nurse practitioner groups after 10 months (Table 1).

### 2.4. Survey Development and Administration

The UCLA Interprofessional Pediatric Oral Health Survey (UCLA IPOH-S) is a 23-item instrument developed by the authors to assess changes in attitudes, skills, and knowledge of pediatric oral health. Knowledge was evaluated by 10 open-ended questions to avoid obtaining the correct answer by chance. The open-ended questions were scored as incorrect [0 points], partially correct [1 point], or correct [2 points] by a single oral health expert with 100 percent inter-rater agreement by a second expert on five surveys. The survey underwent content validity by an expert panel of three dental curriculum experts, with edits made per reviewer recommendations prior to dissemination. The UCLA IPOH-S was accessed using the web-based platform Survey Monkey^®^ [15] and took approximately 7–10 min to complete (see Supplementary Materials).

### 2.5. End of Program Focus Groups

One trained professional focus group facilitator conducted all nine focus groups to maintain standardization. Focus groups lasted 30 min each and were performed separately for the PNP, FNP, and pediatric medicine resident cohorts at the end of the oral health education program. Two focus groups were conducted for the PNP students (n = 13, n = 10), five for the FNP students due to the large cohort (n = 12, n = 9, n = 11, n = 7, n = 3), and two for the pediatric medicine residents (n = 12, n = 11). The focus groups were audio recorded and performed by a professional facilitator using a semi-structured guide with six open-ended questions developed by the CARE-PD program administrators. Questions addressed the usefulness of the oral health training, skills acquired, preparedness for the oral health needs in vulnerable or special needs populations, interprofessional experiences, and suggestions for improving the oral health education training for future cohorts. Additional probing questions were used to clarify and expand on responses. Participation was voluntary, and no monetary compensation was provided to the students for participation.

### 2.6. Statistical Analysis

Survey data were exported to an Excel spreadsheet and converted into a Statistical Package for the Social Sciences (SPSS; Version 28)-formatted data set [16]. Descriptive statistics were used to assess the distribution of demographic characteristics with means and standard deviations for continuous and Chi-squared for categorical variables. A paired sample *t*-test was used to assess differences in mean knowledge scores. The McNemar–Bowker and Marginal Homogeneity test were used to assess differences in attitudes and skills (categorical variables with >2 categories) pre- and post-educational curriculum. Thematic analyses were performed by the project director using Atlas.ti 22 [17] with the focus group transcripts to identify common responses, ideas, or patterns of meaning that repeatedly emerged among participants.

## 3. Results

### 3.1. Survey Completion and Sample Characteristics

The pre- and post-survey completion percentage was 61% for the pediatric medicine residents [14 out of 23], 72% for the PNP group [18 out of 23], and 89% for the FNP group [49 out of 55]. A total of 81 participants who completed both pre- and post-surveys were included in the final analyses. The sample characteristics included 14 pediatric medicine residents (mean age 28.8 [1.5], 50% White, 80% with good to excellent oral health), 18 PNP (mean age 28.7 [3.3], 39% White, mean registered nurse experience 4.5 years [2.4], 89% with good to excellent oral health) and 49 FNP students (mean age 30 [6.2], 35% White and 35% Asian, mean registered nurse experience 5.4 years [3.6], and 99% with good to excellent oral health) (Table 1).

### 3.2. Survey Response

The survey identified no statistically significant differences in personal attitudes regarding oral hygiene (e.g., brushing, flossing, using fluoride toothpaste, and visiting the dentist) pre- and post-educational curriculum, except the PNP group showed improvement in the frequency of dental visits from once a year to twice a year (*p* = 0.027) (Table 1).

The survey showed improved skills, more so in the nurse practitioner groups related to routine oral exams as part of clinical practice (PNP [*p* = 0.002] and FNP [*p* = 0.021]), providing preventative dental education (PNP [*p* < 0.001] and FNP [*p* < 0.001]), and assisting families with dental referrals (PNP [*p* = 0.001] and FNP [*p* < 0.001]). The use of a caries risk assessment form was significant across all groups (pediatric resident [*p* = 0.002], PNP [*p* = 0.001], FNP [*p* < 0.001]). The opportunity to apply fluoride varnish was not significant across all groups, with the pediatric resident group applying more than the nurse practitioner groups (Table 1).

Overall, knowledge scores showed statistically significant improvement in pre- and post-educational curriculum across all groups (pediatric resident = 7.86 [2.4) vs. 14.0 [2.9], *p* < 0.001; PNP = 7.61 [2.7] vs. 15.8 [2.7], *p* < 0.001; FNP = 5.48 [2.8] vs. 12.6 [3.0], *p* < 0.001), respectively. The PNP group had the biggest percent change in pre- and post-educational curriculum scores (8.19%), followed by the FNP (7.12%), and pediatric resident (6.14%) groups (Table 2).

### 3.3. Qualitative Responses

The focus group themes and supporting quotes from the pediatric medicine residents and PNP and FNP students are listed in Table 3. The pediatric medicine residents, PNP, and FNP groups felt the *Smiles for Life* modules were informative, visually helpful in identifying caries, and easy to comprehend. However, some FNP and pediatric residents felt the modules and paper were too much additional work and would have preferred the content be provided in a lecture or simulation (e.g., caries risk assessment). Comments on knowledge learned that could be applied in their clinical practice resonated among participants (e.g., signs and symptoms of caries, and anticipatory guidance across the life span). All groups expressed enjoyment of the IPE portions of the program, peer-to-peer learning from the dental fellows regarding fluoride varnish application/simulation lab, child positioning for oral exams, and group discussions.

## 4. Discussion

Our study findings showed that the CARE-PD IPE curriculum significantly improved oral health knowledge across all groups of providers based on survey responses to pediatric oral health questions. The physician group had higher pre-knowledge scores, which may reflect that they were in their second year of residency and had more pediatric clinical experience than first-year PNP and FNP students. FNP students often have little to no pediatric nursing experience before the start of their nurse practitioner program, which could explain their group having the lowest pre-knowledge score compared to the PNP students, where pediatric nursing experience is required.

Other studies of IPE programs have produced similar findings related to improved knowledge across multiple health science students from dentistry, nursing, medicine, pharmacy, and public health [9,18,19]. However, curriculum content integrated into academic programs, the duration of IPE programs, and outcome measures or instruments used varied across both U.S. and European studies [5,9]. In our program, 11 h of the CARE-PD curriculum was integrated over three quarters or one academic year for the PNP and FNP groups, and the content was placed in courses with similar curricula. For example, the FNP and PNP groups included the oral exam *Smiles for Life* module in their respective advanced physical assessment course. In the PNP group, their growth and development course already contained pediatric oral health content, so the child oral health and caries risk assessment modules and didactic lectures were included in this course. In addition, the FNP’s required women’s health course included the pregnancy and oral health module. On the other hand, the pediatric medicine residence had the CARE-PD curriculum delivered over eight consecutive weeks. With the curriculum spaced out over a 30-week academic year or provided in a shorter time frame of eight weeks, improved oral health knowledge was seen in all groups. One study, which provided a 10-week IPE course on pediatric oral health to dental, osteopathic medical, and nurse practitioner students, showed overall improved knowledge and confidence in the students’ ability to provide oral health services [19]. Since the beginning of our oral health curriculum for medical/dental integration ten years ago, we have seen the importance of ongoing oral health training for the retention of health professionals’ knowledge and their awareness of the importance of oral health in their patients’ overall health. One of the challenges to IPE education is the coordination of multiple health sciences programs to meet and the lack of space to add 10–12 h of additional content to an established curriculum. Spreading the modules out over three quarters for the nurse practitioner students allowed us to thread content in like courses to highlight the oral-systemic connection in vulnerable populations or clinical conditions. Many qualitative comments from the nurse practitioner-focus groups supported this dissemination of the curriculum versus being “jammed into one lecture on one day.” The pediatric medicine resident group did not express dissatisfaction with completing the curriculum over eight weeks; antidotal comments reflected wanting more hands-on experiences, resources for parents, and more information on dental providers for underserved Medicare patients.

Improved clinical skills were seen in the nurse practitioner groups more than in the physician group. Pre-skills assessment showed that many physician providers were already performing oral exams, providing oral health anticipatory guidance, dental home referrals, and applying fluoride varnish in the clinical setting. However, the increased use of a caries risk assessment tool in clinical practice was significant across all providers. Previous studies have identified that pediatric providers commonly perform dental screening or exams, but few use a caries risk assessment tool [20]. Risk assessment tools can aid in the identification of reliable predictors and allow dental practitioners and other non-dental healthcare providers to identify and refer high-risk children [21]. Our findings were encouraging that providers felt caries risk assessments were helpful and showed increased usage. The relevance of having providers perform regular caries risk assessments shows an incredible accomplishment in bringing oral health to their regular practice as a standard of care.

There was no improvement in attitudes about oral hygiene except in the PNP group, who increased their visits to the dentist from once to twice a year. Other dental IPE programs had similar findings but defined attitudes differently, such as participants’ attitudes toward providing children with oral health care [19]. Our survey examined attitudes and frequency of personal oral hygiene (e.g., brushing, flossing, fluoride, and dental visits) and whether improvement occurred after the IPE program. The lack of improvement may reflect highly educated students with good baseline oral hygiene practices, as evidenced by the survey participants’ ratings of good to excellent oral health.

Students who have completed IPE programs have reported higher self-efficacy in functioning as a member of an interdisciplinary team [22,23]. The IPE SPICE-PD program demonstrated a positive effect on IPE experiences and collaboration of care [7]. Furthermore, oral health IPE programs with nurse practitioner students provided adequate knowledge, awareness, confidence, and attitude regarding oral health [13,24,25].

Since children will often see a medical provider more than a dentist for preventative care in the first 3 years of life [26], the public health impact of training non-dental healthcare providers is crucial in combating ECC. The CARE-PD IPE program could be used as a model for other IPE training programs. Future research is needed to assess the state-level impact of increased numbers of trained providers in the community on pediatric oral health.

### 4.1. Limitations

Although this is a unique IPE program, there were limitations to the study results, with potential impact on validity. The survey was developed by the IPE team, the sample size was small, with no control group, and reflected a single center, which limits the generalizability of our findings. Though residents and nurse practitioner students were instructed not to, some could have looked up the answers during the pre-knowledge and post-knowledge assessment surveys. The medicine residents and some nurse practitioner students may have had more clinical experience before the start of the program or had more exposure than others to oral health. The time frame for the curriculum was not standardized across programs. This may account for the lack of percent change and significant differences in pre- and post-survey responses in the medicine resident group.

### 4.2. Implications for Future Research

Essential to all professional health education programs is the need to anticipate and adapt to changing healthcare needs. In the future, academic programs must develop an oral health program or emphasize more dental content as part of their overall curriculum to create a complete medical/dental integration. Training all faculty in the academic setting to adapt to the new curriculum/content threaded through workflow adaptation is essential. Learner feedback is critical to the success of the integration process. With emerging oral health data, practices to facilitate oral health integration, and routine health care, there is a need for new educational strategies to better support the comprehension and retention of oral health concepts. The core competencies of IPE should be grounded by the Interprofessional Education Collaborative Expert Panel (IPEC) to provide the foundation for preparing all health professionals [27,28].

## 5. Conclusions

The successful integration of oral health curriculum into medicine and nurse practitioner program curriculum can be challenging and multifaceted. Healthcare providers must acquire knowledge and skills to conduct oral health assessments and offer preventive advice and initial management, including dental referrals. This was accomplished through the IPE CARE-PD curriculum between UCLA dentistry, medicine, and nursing schools. With shared best practices for oral health curriculum integration and program evaluations, we can effectively teach about oral health, have providers deliver more complete care, and ultimately provide young children with better, more equitable care to mitigate ECC. Future studies should focus on the adoption of core curriculum and validating evaluation measures for the many types of non-dental healthcare providers.

## Figures and Tables

**Table 1:** Integration of the oral health curriculum into medicine and nurse practitioner programs.

Curriculum	Pediatric Medicine	PNP Students—1st Year			FNP Students—1st Year			Evaluation
Academic Quarters	Winter—8 Weeks	Fall	Winter	Spring	Fall	Winter	Spring	
Pre-Knowledge	X *	X **			X **			SurveyMonkey^®^
*Smiles for Life* Modules [1 h each]								Module Questions and Certificate of Completion
1 = Oral to Systemic Health	X	X						
2 = Child Oral Health	X				X		X	
5 = Pregnancy Oral Health	X		X			X		
6 = CRA	X			X			X	
7 = The Oral Exam	X	X	X			X		
Didactic [Interdisciplinary; 5 h]								CRA Practice & Written Assignment with Anticipatory Guidance
Oral Health Lecture			X			X		
CRA Lecture	X		X			X		
Dental Trauma Brown Bag				X			X	
Skills Lab [Dentistry Fellows; 1 h]	X							Peer–Peer Learning Simulation
Oral exam positioning				X			X	
Fluoride Varnish Application				X			X	
End-of-Program Evaluation	X			X			X	Focus Group SurveyMonkey®
Post-Knowledge	X *			X **			X **	

CRA = caries risk assessment; X = timepoint when activity performed; * 8 week gap, ** 10 month gap between pre- and post-knowledge survey.

**Table 2:** Sample characteristics, attitudes, skills, and knowledge pre- and post-CARE-PD by pediatric medical residents, pediatric and family nurse practitioner students.

Variables.	Pediatric Medical Residents [n = 14]			Pediatric NP Students [n = 18]			Family NP Student [n = 49]		
**Characteristics**	**n (%) or Mean [SD]**								
Age, years	28.8 [1.5] (range 27–32)			28.7 [3.4] (range 24–38)			30.7 [6.2] (range 23–49)		
Ethnicity									
White	7 (50%)			7 (39%)			17 (35%)		
Hispanic	3 (21%)			4 (22%)			13 (26%)		
Asian	3 (21%)			6 (33%)			17 (35%)		
Black	1 (8%)			1 (6%)			2 (4%)		
RN Experience, years	N/A			4.5 [2.4] (range 2–13)			5.4 [3.7] (range 1–22)		
General Oral Health									
Excellent	3 (21%)			4 (22%)			10 (20%)		
Very Good	2 (21%)			10 (56%)			13 (28%)		
Good	5 (37%)			2 (11%)			25 (50%)		
Fair	3 (21%)			2 (11%)			1 (2%)		
**Attitudes**									
	**Pre**	**Post**	***p***	**Pre**	**Post**	***p***	**Pre**	**Post**	***p***
**How often do you brush your teeth?**									
Once daily	2 (14%)	2 (14%)		0 (0%)	1 (6%)		3 (6%)	3 (6%)	
>2 × a daily	12 (86%)	12 (86%)	1.00	18 (100%)	17 (94%)	1.00	45 (92%)	45 (92%)	1.00
Every other day	0 (0%)	0 (0%)		0 (0%)	0 (0%)		1 (2%)	1 (2%)	
<3 × per week	0 (0%)	0 (0%)		0 (0%)	0 (0%)		0 (0%)	0 (0%)	
**Do you use fluoride toothpaste?**			0.366			1.00			0.425
Yes	11 (79%)	13 (93%)		16 (88%)	16 (88%)		37 (76%)	40 (82%)	
No	1 (7%)	0 (0%)		1 (6%)	1 (6%)		4 (8%)	3 (6%)	
Unsure	2 (14%)	1 (7%)		1 (6%)	1 (6%)		8 (16%)	6 (12%)	
**How often do you floss your teeth?**			0.223			0.102			0.655
Once daily	9 (65%)	11 (79%)		9 (50%)	11 (61%)		26 (53%)	29 (60%)	
Once a week	2 (14%)	1 (7%)		6 (33%)	2 (11%)		13 (27%)	11 (22%)	
1–2 × month	2 (14%)	1 (7%)		3 (17%)	3 (17%)		8 (16%)	7 (14%)	
I do not floss	1 (7%)	1 (7%)		0 (0%)	2 (11%)		2 (4%)	2 (4%)	
**How often do you visit the dentist?**			0.414			0.027			0.180
>2 × per year	3 (22%)	7 (50%)		3 (17%)	13 (71%)		27 (55%)	29 (59%)	
Once a year	7 (50%)	6 (43%)		12 (67%)	3 (17%)		16 (33%)	16 (33%)	
Every 2–3 years	2 (14%)	1 (7%)		1 (6%)	1 (6%)		2 (4%)	2 (4%)	
Only if problem	2 (14%)	0 (0%)		2 (10%)	1 (6%)		4 (8%)	2 (4%)	
**Skills**									
	**Pre**	**Post**	***p***	**Pre**	**Post**	***p***	**Pre**	**Post**	***p***
**Is an oral exam part of your routine clinical practice?**			0.197			0.002			0.021
Most of the time	4 (29%)	4 (29%)		1 (6%)	10 (56%)		10 (20%)	8 (16%)	
Some of the time	4 (29%)	8 (57%)		6 (33%)	6 (33%)		11 (22%)	26 (53%)	
Rarely	5 (35%)	2 (14%)		10 (55%)	2 (11%)		19 (40%)	11 (22%)	
Never	1 (7%)	0 (0%)		1 (6%)	0 (0%)		9 (18%)	4 (9%)	
**Do you use a caries risk assessment form in your clinical practice?**			0.002			0.001			<0.001
Most of the time	0 (0%)	1 (7%)		0 (0%)	4 (22%)		0 (0%)	3 (6%)	
Some of the time	0 (0%)	3 (21%)		1 (6%)	6 (33%)		0 (0%)	6 (12%)	
Rarely	0 (0%)	7 (51%)		2 (11%)	5 (28%)		8 (16%)	13 (27%)	
Never	14 (100%)	3 (21%)		15 (83%)	3 (17%)		41 (84%)	27 (55%)	
**Do you provide preventative dental education at pediatric visits?**			0.782			<0.001			<0.001
Most of the time	11 (79%)	10 (71%)		1 (6%)	15 (83%)		2 (4%)	9 (18%)	
Some of the time	2 (14%)	4 (29%)		3 (17%)	2 (11%)		3 (6%)	11 (22%)	
Rarely	0 (0%)	0 (0%)		4 (22%)	0 (0%)		8 (16%)	10 (20%)	
Never	1 (7%)	0 (0%)		10 (55%)	1 (6%)		36 (74%)	19 (40%)	
**Do you assist families with referrals to dentists if needed?**			0.593			0.001			<0.001
Most of the time	10 (72%)	10 (72%)		0 (0%)	9 (50%)		2 (4%)	8 (16%)	
Some of the time	3 (21%)	4 (28%)		6 (33%)	4 (22%)		7 (14%)	15 (31%)	
Rarely	0 (0%)	0 (0%)		2 (11%)	4 (22%)		10 (20%)	15 (31%)	
Never	1 (7%)	0 (0%)		10 (56%)	1 (6%)		30 (62%)	11 (22%)	
**Have you had the opportunity to apply fluoride varnish in your clinical practice?**			0.416			0.058			0.239
Most of the time	7 (50%)	6 (44%)		0 (0%)	1 (6%)		0 (0%)	2 (4%)	
Some of the time	1 (7%)	3 (21%)		0 (0%)	1 (6%)		1 (2%)	1 (2%)	
Rarely	1 (7%)	2 (14%		1 (6%)	2 (11%)		2 (4%)	2 (4%)	
Never	5 (36%)	3 (21%)		17 (94%)	14 (77%)		46 (94%)	44 (90%)	
**Knowledge**									
	**Pre**	**Post**	***p***	**Pre**	**Post**	***p***	**Pre**	**Post**	***p***
Scores, mean Total (0–20)	7.86 [2.4]	14.0 [2.9]	<0.001	7.61 [2.7]	15.8 [2.7]	<0.001	5.48 [2.8]	12.6 [3.0]	<0.001
Change in Score (%)	6.14%			8.19%			7.12%		

**Table 3:** Focus group themes and supporting quotes from pediatric medicine residents and pediatric and family nurse practitioner students.

Pediatric Medicine Residents (N = 23)	
**Themes**	**Supporting Quotes**
Attitudes towards the oral health training and *Smiles for Life* modules.	“*Smiles for Life* was really helpful. I thought that it was a great website to go to for resources too. It’s definitely something I’ll come back to when I need to look stuff up for my practice.” “I thought the photos [in *Smiles for Life*] were great…having a visual to take a look at, like, different tooth injuries and infections and was kind of a good clinical pearl to see.” “I think the modules were also very easily digestible—like we weren’t inundated with information, it was very brief, to the point, and exactly what we would need to know as pediatricians.”
Application of the oral health training to current practice.	“The knee-to-knee exam I’ve been able to do a couple times and that’s been nice.” “So, I think it was good just to see what questions the dentists are asking and then which ones we can adapt to our practice, especially for the kids who don’t have a dental home which is a lot of our clinic patients.” “In terms of applying the fluoride, it was nice to see the strategies recommended to use…. So just learning specifically how they recommend, like, holding the child and then where on the teeth to start. I feel like that specifically was helpful.”
Suggestions for program improvement.	“Making it more interactive and maybe even, you know, have a group of 10 residents we can break up into groups of three and then each practice more hands-on so that I kind of remember what to do when I’m in clinic versus the picture portion.” “Maybe if there were resources we could share with parents, like frequently asked questions or handouts or something like that, like something tangible we could get.” “Another thing I feel like we could’ve talked more about is how to help out patients who are underserved or vulnerable get access to dental care. Like talking more about the insurance situation when it comes to dental care or how available pediatric dentist who accept Medicare are, things of that nature.”
**Pediatric Nurse Practitioners (N = 23)**	
**Themes**	**Supporting Quotes**
Application of the oral health training to current practice.	“I’ve gotten to apply like varnish before, and it was at a clinic where it wasn’t like standard practice to do that and because of the training, I kind of showed other providers how easy it was.” “On all my Well-child visits, I check their teeth and I look for white spot lesions and then I’ll say like, “Do you eat candy?” “Are you brushing your teeth?” And normally the answer is no, and so then we get to talk about that.” “Yesterday I had a two-month-old, and the parents didn’t realize that they were supposed to gently wipe down the gums, like in between feedings or like at least try to do it once a day, at the very least, so I was able to educate them on oral care.”
Attitudes towards the oral health training and *Smiles for Life* modules/lectures.	“I thought it was really helpful, I never felt like we got proper dental education in nursing school.” “I kind of liked that we just got to do some modules on our own time. I think the modules were set up like, well—that you like go through, you take a quiz, and it helps you kind of just remember these little highlighted facts.” “So, like the modules gave me such good visual reference that now I look at kids and don’t just think like, “Okay, they’ve got some weird teeth.” But like now I can like actually look at their teeth and be like, “Okay, they’re this far into like having a cavity.” Or some plaque formation or if there’s something like really—give like guidance and be able to like accurately explain that to families gives like you more confidence.” “I think one strength is that this is like how we bridge the gap between like dentistry and medical care, because I really feel like there is a big disconnect between those two. But now I feel like there’s more of a connection because I understand what happens on the other side and what I can do to promote that.”
Interprofessional experiences during the oral health training program.	“They’re just like knowledgeable and they really, I think, also understood our perspective, like even the dentist that lectured for us like he was trying to make it very understanding from the primary care perspective.” “They’ve [dentists] all been very willing to teach and explain things in a way that is applicable for us and like easy to understand, but also helped me understand the relevance too.” “To get it [information] from somebody that is a dentist coming from our program, being able to tell us like “Here if you need it.” I think that was beneficial.”
Barriers to conducting a caries risk assessment.	“Time constraints.” “With the old school doctors, there’s not really a lot of emphasis on the dental stuff, so they don’t necessarily prioritize it.” “I think, with like our clinic, our kids are so complex with like DCFS involvement and things like that and such low resources already that sometimes dental and oral health gets kind of put on the back burner if we’re just trying to get them vaccinated… or up to their goal weight... So, those things can take up the whole time that we have with them and the least of our worries sometimes can be their teeth, you know.”
**Family Nurse Practitioners (N = 42)**	
**Themes**	**Supporting Quotes**
Program strengths.	“I think one thing for me was the link between oral health and systemic health. I think that was in the very—one of the very first like *Smiles for Life* modules that we did was like, the link between like oral health and systemic inflammation and kind of how everything kind of plays into each other.” “One thing that really stuck out to me was just learning that kids should be going to see a dentist as early as like their first tooth eruption or the first year of life, which I hadn’t really realized.” “For me it was the caries risk assessment and then just like the risk of caries that can have on kids and then feeling empowered that I could actually do something aside from referring to the dentist in clinic, which is the varnish application.” “Being able to apply varnish and being taught how to apply varnish. I thought that that was really helpful as well in improving the outcomes for our patients.” “It’s kind of nice that it’s spread out across multiple quarters instead of just being jammed into one lecture on one day. So, it’s just kind of like little bites at a time.”
Interprofessional experiences during the oral health training program.	“I really appreciated having the interdisciplinary interaction with a dentist at our simulation lab. But I wish we had more interaction with the dentistry school during this, especially in a small group setting, because I think that would help clarify the information and help us bridge that gap that we often see between the medical world and the dental world.” “I think just through nursing experience and watching physicians, sometimes physician specialties or other specialties don’t want you to touch their area, and so, I found it really helpful to have actual input and feedback and training from the dentist.” “I think there’s a lot of myths that, you know, primary care providers and dentists don’t really speak to each other or refer. And so, I think it was really good to meet the dentist and you know, make sure that we’re all on the same page, and that’s really to help our patients and prevent any diseases that may come up with, with oral care, dental care and things like that.”
Suggestions for program improvement.	“If we could cut down on that online curriculum and then have like one hour-long in-person thing per quarter over the first year instead of the online stuff. I think it would probably like stick better, honestly.” “If maybe we were able to do the caries risk assessment in a simulation in class, I think that would’ve been more appreciated by us just because we all like the simulation’s lab, versus like sitting down and writing a paper, which, you know—I think we would just retain better if we had the in-person visual audio and tactile.” “Just the timing of the actual modules, definitely makes a huge difference. Being more aligned with our pediatrics class and then, maybe having us do them like at the very beginning of the quarter when we don’t have a ton of other work to do at the time might be helpful.”

## Data Availability

The authors declare their willingness to share de-identified data that support the findings of this study and are available upon request from the corresponding authors; data-use agreements will be needed to be completed and approved to share data by the University of California, Los Angeles.

## Supplementary Materials

The following supporting information can be downloaded at: https://www.mdpi.com/article/10.3390/healthcare12181807/s1, UCLA Interprofessional Pediatric Oral Health Questionnaire [UCLA IPOH-Q].
