# Supplementary material for: Oral Health Knowledge, Attitudes, and Learned Clinical Skills in Pediatric Medicine Residents and Nurse Practitioner Students: A Pre-Post Design
Source: Healthcare (Basel). 2024 Sep 10;12(18):1807. doi: 10.3390/healthcare12181807 (PMC11431633; doi:10.3390/healthcare12181807)
Supplement: Supplementary file 1 [file healthcare-12-01807-s001.zip › healthcare-3144570-supplementary.pdf]

Supplementary Material: UCLA Interprofessional Pediatric Oral Health Questionnaire [UCLA IPOH-Q]

Instructions: The UCLA IPOH-Q is a **brief 20-item** questionnaire designed to assess your knowledge of pediatric preventative dentistry from **age 0 to 5 years**. Please read each question carefully and write what you consider to be correct. Please answer all questions to the best of your knowledge.

Professional Type
[ ] Pediatric Nurse Practitioner Student
[ ] Family Nurse Practitioner Student
[ ] Pediatric Medicine Resident

Age: ____________ years

Ethnicity:
[ ] White / Caucasian
[ ] Hispanic
[ ] African American
[ ] Asian / Pacific Islander
[ ] Other _____________________

RN Experience: _____________ years

Self-Rated Dental Health
In general, would you say your dental health is?
[ ] Excellent
[ ] Very Good
[ ] Good
[ ] Fair
[ ] Poor

Attitudes –
The following are questions about your current dental hygiene practices.

1. How often do you brush your teeth?
a. Two or more times per day
b. Once daily
c. Once every other day
d. Less than 3 times per week

2. Do you use fluoride toothpaste?
a. Yes
b. No
c. Sometimes
d. Unsure

3. How often do you floss your teeth?
a. Once daily
b. Once a week
c. One to two times a month
d. I do not floss my teeth

4. How often do you visit a dentist?
a. Twice or more per year
b. Once per year
c. Every two or three years
d. Only when I have an issue with my teeth

5. What are the types of foods that you snack on in between meals?
a. Protein Bars
b. Chips, Crackers or Cookies
c. Fruit or Vegetables
d. Nuts and Seeds

Skills

The following questions are about your current clinical practice and incorporating dental exams and pediatric preventative care.

1. **Is an oral exam of teeth and gums part of your routine clinical practice?**

a. Most of the time
b. Some of the time
c. Rarely
d. Never

2. **Do you use a caries risk assessment form (e.g. AAP, CAMBRA) in your clinical practice?**

a. Most of the time
b. Some of the time
c. Rarely
d. Never

3. **Do you currently provide preventative dental education as part of anticipatory guidance at pediatric visits?**

a. Most of the time
b. Some of the time
c. Rarely
d. Never

4. **Do you take the opportunity to assist families with referrals to dentists in the community if appropriate?**

a. Most of the time
b. Some of the time
c. Rarely
d. Never

5. **Have you had the opportunity to apply fluoride varnish in your clinical practice?**

a. Most of the time
b. Some of the time
c. Rarely
d. Never

Knowledge

The following questions are to assess your current knowledge about pediatric preventative dentistry.

1. All primary teeth should erupt by what age?
2. At what age should the first pediatric dental visit occur?
3. How much toothpaste does a child < 3 years of age require for brushing?
4. How often should a child have a dental check-up?
5. What is the most common mode of transmission of bacteria from mother to infant that can cause early dental caries?
6. Describe the best way to perform an oral exam on a young child?
7. List 3 clinical findings that places a child at increase caries risk.
8. How often should fluoride varnish application be done on children at high-risk for ECC (early childhood caries)?
9. List dental treatments that are safe during pregnancy?
